# 26. Risk of Post–COVID-19 Dyspnea and Interstitial Lung Disease (ILD) in a Real-World Cohort of Patients Hospitalized with COVID-19 in the United States

**DOI:** 10.1093/ofid/ofab466.026

**Published:** 2021-12-04

**Authors:** Kelly Zalocusky, Devika Chawla, Margaret Neighbors, Shemra Rizzo, Larry Tsai

**Affiliations:** Genentech, Inc., South San Francisco, California

## Abstract

**Background:**

While COVID-19 carries substantial morbidity and mortality, the extent of long-term complications remains unclear. Reports suggest that acute lung damage associated with severe COVID-19 can result in chronic respiratory dysfunction. This study: (1) estimated the incidence of dyspnea and ILD after COVID-19 hospitalization, and (2) assessed risk factors for developing dyspnea and ILD in a real-world cohort of patients hospitalized with COVID-19 using US electronic health records (EHR).

**Methods:**

Patients in the Optum de-identified COVID-19 EHR database who were hospitalized for COVID-19 (lab confirmed or diagnosis code) between February 20 and July 2020 and had at least 6 months of follow-up were eligible for analysis. Dyspnea and ILD were identified using diagnosis codes. The effects of baseline characteristics and hospitalization factors on the risk of incident dyspnea or ILD 3 to 6 months’ post discharge were evaluated.

**Results:**

Among eligible patients (n=26,339), 1705 (6.5%) had dyspnea and 220 (0.8%) had ILD 3 to 6 months after discharge. Among patients without prior dyspnea or ILD (n=22,613), 110 (0.5%) had incident ILD (**Table 1**) and 1036 (4.6%) had incident dyspnea (**Table 2**) 3 to 6 months after discharge. In multivariate analyses, median (IQR) length of stay (LOS; 5.0 [3.0, 9.0] days in patients who did not develop ILD vs 14.5 [6.0, 26.0] days in patients who developed ILD; RR: 1.12, 95% CI: 1.08, 1.15; *P*=4.34 x 10^-10^) and age (RR: 1.02, 95% CI: 1.01, 1.03; *P*=4.63 x 10^-3^) were significantly associated with ILD. Median (IQR) LOS (5.0 [3.0, 9.0] days in patients who did not develop dyspnea vs 7 [4.0, 14.0] days in patients who developed dyspnea; RR: 1.04, 95% CI: 1.02, 1.06; *P*=8.52 x 10^-4^), number of high-risk comorbidities (RR: 1.18, 95% CI: 1.12, 1.24; *P*=3.85 x 10^-9^), and obesity (RR: 1.52, 95% CI: 1.25, 1.86; *P*=2.59 x 10^-4^) were significantly associated with dyspnea.

Table 1. Selected Baseline Risk Factors for Incident ILD

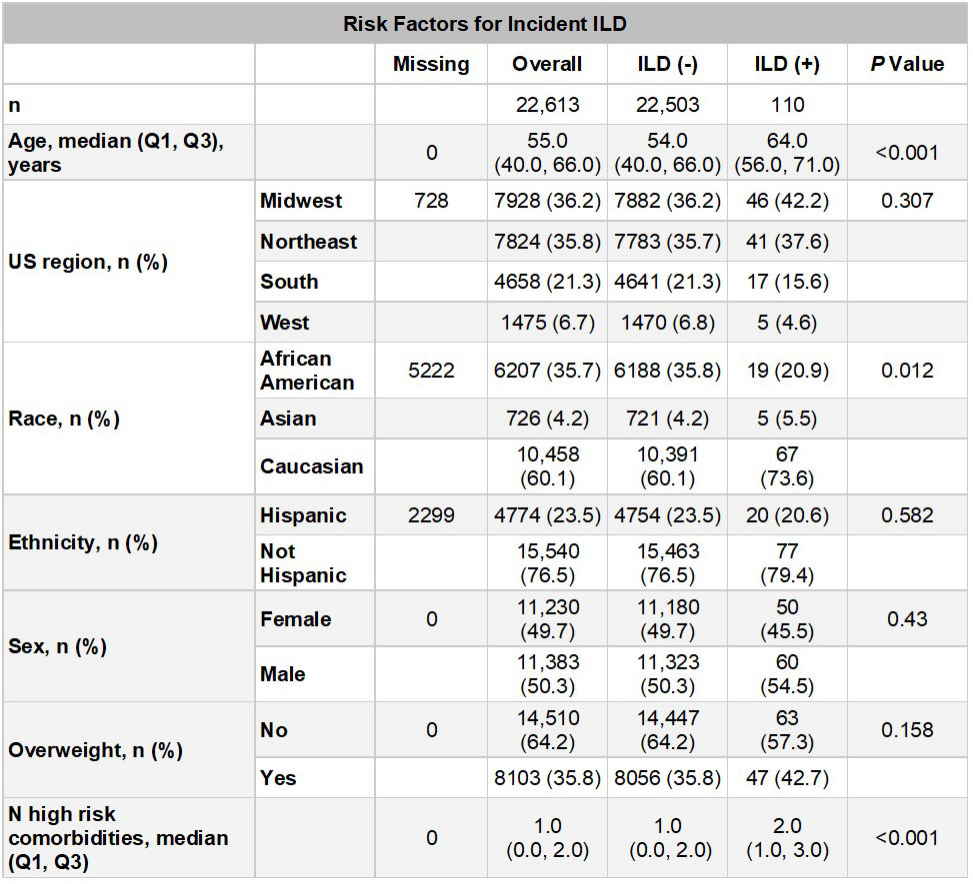

Table 2. Selected Baseline Risk Factors for Incident Dyspnea

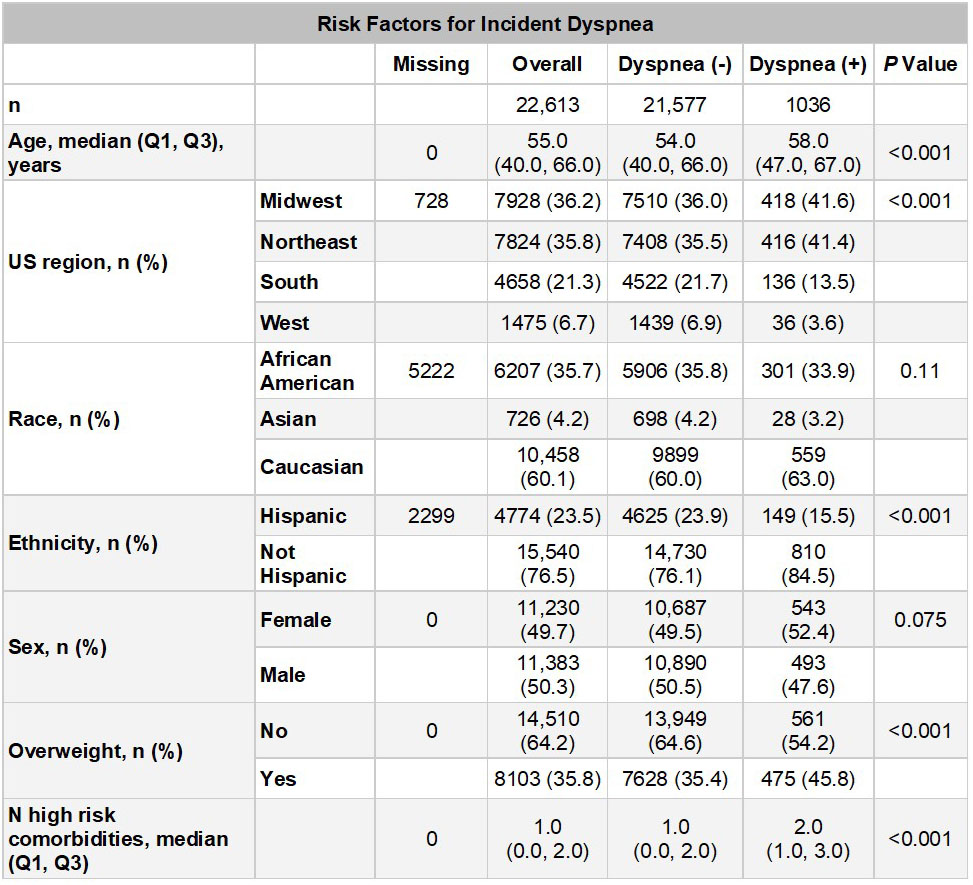

**Conclusion:**

In a real-world cohort, 4.6% and 0.5% of patients developed dyspnea and ILD, respectively, after COVID-19 hospitalization. Multivariate analyses suggested that LOS, age, obesity, and comorbidity burden may be risk factors for post-COVID-19 respiratory complications. Limitations included sensitivity of diagnosis codes, availability of labs, and care-seeking bias.

**Disclosures:**

**Kelly Zalocusky, PhD**, **F. Hoffmann-La Roche Ltd** (Shareholder)**Genentech, Inc.** (Employee) **Devika Chawla, PhD MSPH**, **F. Hoffmann-La Roche Ltd.** (Shareholder)**Genentech, Inc.** (Employee) **Margaret Neighbors, PhD**, **F. Hoffmann-La Roche Ltd** (Shareholder)**Genentech, Inc.** (Employee) **Shemra Rizzo, PhD**, **F. Hoffmann-La Roche Ltd.** (Shareholder)**Genentech, Inc.** (Employee) **Larry Tsai, MD**, **F. Hoffmann-La Roche Ltd** (Shareholder)**Genentech, Inc.** (Employee)

